# Functional analysis of *Arabidopsis *WRKY25 transcription factor in plant defense against *Pseudomonas syringae*

**DOI:** 10.1186/1471-2229-7-2

**Published:** 2007-01-10

**Authors:** Zuyu Zheng, Stephen L Mosher, Baofang Fan, Daniel F Klessig, Zhixiang Chen

**Affiliations:** 1 Department of Botany and Plant Pathology, Purdue University, West Lafayette, IN 47907-2054, USA; 2 Boyce Thompson Institute for Plant Research, Tower Road, Cornell University, Ithaca, NY 14853, USA

## Abstract

**Background:**

A common feature of plant defense responses is the transcriptional regulation of a large number of genes upon pathogen infection or treatment with pathogen elicitors. A large body of evidence suggests that plant WRKY transcription factors are involved in plant defense including transcriptional regulation of plant host genes in response to pathogen infection. However, there is only limited information about the roles of specific WRKY DNA-binding transcription factors in plant defense.

**Results:**

We analyzed the role of the WRKY25 transcription factor from *Arabidopsis *in plant defense against the bacterial pathogen *Pseudomonas syringae*. WRKY25 protein recognizes the TTGACC W-box sequences and its translational fusion with green fluorescent protein is localized to the nucleus. *WRKY25 *expression is responsive to general environmental stress. Analysis of stress-induced *WRKY25 *in the defense signaling mutants *npr1*, *sid2*, *ein2 *and *coi1 *further indicated that this gene is positively regulated by the salicylic acid (SA) signaling pathway and negatively regulated by the jasmonic acid signaling pathway. Two independent T-DNA insertion mutants for *WRKY25 *supported normal growth of a virulent strain of *P. syringae *but developed reduced disease symptoms after infection. By contrast, *Arabidopsis *constitutively overexpressing *WRKY25 *supported enhanced growth of *P. syringae *and displayed increased disease symptom severity as compared to wild-type plants. These *WRKY25*-overexpressing plants also displayed reduced expression of the SA-regulated *PR1 *gene after the pathogen infection, despite normal levels of free SA.

**Conclusion:**

The nuclear localization and sequence-specific DNA-binding activity support that WRKY25 functions as a transcription factor. Based on analysis of both T-DNA insertion mutants and transgenic overexpression lines, stress-induced WRKY25 functions as a negative regulator of SA-mediated defense responses to *P. syringae*. This proposed role is consistent with the recent finding that WRKY25 is a substrate of *Arabidopsis *MAP kinase 4, a repressor of SA-dependent defense responses.

## Background

Plants are subjected to constant attack by a variety of microbial pathogens and herbivores and they have evolved a complex battery of defense mechanisms that are activated by multiple defense signaling pathways. Thus, in response to infection by some microbial pathogens, the interacting EDS1 and PAD4 proteins are induced and activated; these proteins positively regulate biosynthesis of salicylic acid (SA) and SA-dependent defense signaling pathways [1]. Other defense pathways mediated by ethylene (ET) and jasmonic acid (JA) also can be activated by certain pathogens and herbivores [2,3]. Genetic and molecular analyses indicate that these distinct defense pathways cross-talk extensively and their relationship can be synergistic or antagonistic [4].

Activation of plant defense responses following pathogen infection is associated with induction of a large number of host genes [5]. Some of the pathogen-induced genes encode proteins with direct anti-microbial activities (e.g. the hydrolytic glucanases and chitinases that degrade cell walls of microbial organisms) or enzymes involved in biosynthesis of anti-microbial compounds (e.g. phytoalexins), whereas others encode proteins with regulatory functions in the defense signaling pathways. Many of these defense genes appear to be induced at the transcriptional level through the specific recognition of *cis*-acting promoter elements and *trans*-acting sequence-specific DNA-binding transcription factors. Several classes of transcription factors have been implicated in plant defense responses, including DNA-binding proteins containing the novel WRKY zinc-finger motif [6]. Although originally thought to be plant specific, genes encoding WRKY proteins have been found in two non-photosynthetic eukaryotes, the slime mold *Dictyostelium *and the protist *Giardia lamblia*. The latter two organisms evolutionally precede the divergence of plants from animals and fungi, indicating an ancient origin of WRKY transcription factors [6,7]. However, WRKY genes have greatly proliferated and form large superfamilies in angiosperms, with more than 70 members in *Arabidopsis *[6,7].

A growing body of evidence suggests that WRKY proteins play important roles in regulating genes associated with plant defense responses. For example, pathogen infection or treatment with elicitors or SA rapidly induces WRKY gene expression in several plant species [8-17]. In *Arabidopsis*, expression of many WRKY genes was differentially regulated after pathogen infection or SA treatment [13]. Moreover, many defense-related genes, including several *Pathogenesis-Related *(*PR*) genes and the regulatory *NPR1 *gene, contain W-box elements in their promoters [8,15,18-22]. W-box sequences are specifically recognized by WRKY proteins and are necessary for inducible expression of these genes [8,15,18-22].

In addition to this indirect evidence, several studies have revealed direct links between specific WRKY proteins and plant defense responses. Virus-induced silencing of three *WRKY *genes in tobacco compromised *N *gene-mediated resistance to *Tobacco mosaic virus *[23]. Additionally, the *Arabidopsis *resistance gene *RRS1*, which confers resistance to the bacterial pathogen *Ralstonia solanacearum*, encodes a novel WRKY protein, WRKY52, that combines typical TIR-NBS-LRR R protein motifs with a WRKY domain [24,25]. Moreover, two *Arabidopsis WRKY *genes (*WRKY22 *and *WRKY29*) were shown to be induced by a MAP kinase pathway that mediates resistance responses to both bacterial and fungal pathogens and expression of *WRKY29 *in transiently transformed leaves led to reduced disease symptoms [26]. Likewise, constitutive expression of *Arabidopsis WRKY18 *and *WRKY70 *conferred constitutive or enhanced expression of defense-related genes, including SA-induced *PR1*, and increased resistance to virulent pathogens [27,28]. Interestingly while overexpression of *Arabidopsis WRKY18 *activated SA-regulated *PR1 *gene expression and enhanced resistance to *P. syringae*, its coexpression with genes encoding its interacting partner WRKY40 or WRKY60 had opposite effects on *Arabidopsis *resistance to the bacterial pathogen [29].

In a previously reported study using yeast two-hybrid screening, *Arabidopsis *MAP kinase 4 (MPK4), an activator of JA/ET-mediated defense and a repressor of SA-dependent resistance [30], was found to interact with a MPK4 substrate MKS1 that, in turn, interacts with *Arabidopsis *WRKY25 and WRKY33 [31]. In addition, WRKY25 and WRKY33 were phosphorylated by MPK4 *in vitro *and a *wrky33 *knockout mutant expressed elevated levels of *PR1 *under a short-day growth condition [31]. These results suggest that WRKY25 and WRKY33 may function as downstream components of the MPK4-mediated SA-repressing and JA/ET-activating signaling pathways. Indeed, we have recently shown that disruption of *WRKY33 *results in enhanced susceptibility to necrotrophic fungal pathogens and impaired expression of JA/ET-regulated defense genes [32]. No such phenotypes were observed in the *wrky25 *T-DNA insertion mutants. These results indicate that WRKY33 functions as a positive regulator of JA/ET-mediated pathways and plays an important role in disease resistance to necrotrophic fungal pathogen.

SA-mediated signaling has been shown to play a critical role in *Arabidopsis *responses to infection by *Pseudomonas syringae *[33,34]. We therefore used this model system to investigate the role of WRKY25 in the activation of defense responses. WRKY25 specifically bound the W-box sequence and was localized in the nucleus. In addition, *WRKY25 *is induced by a variety of environmental stress stimuli, including SA and ET treatment. Further linking WRKY25 with defense signaling, transgenic *Arabidopsis *constitutively overexpressing *WRKY25 *displayed reduced pathogen-induced *PR1 *gene expression and enhanced susceptibility to virulent *P. syringae *pv. *maculicola *strain ES4326 (*Psm*ES4326). By contrast, two independent T-DNA insertion mutants for *WRKY25 *developed milder disease symptoms than wild type plants after *Psm*ES4326 infection. These results suggest that WRKY25 plays a negative role in SA-regulated *PR1 *gene expression and resistance to *P. syringae*.

## Results

### Structure, DNA binding and subcellular localization

**Figure 1:**
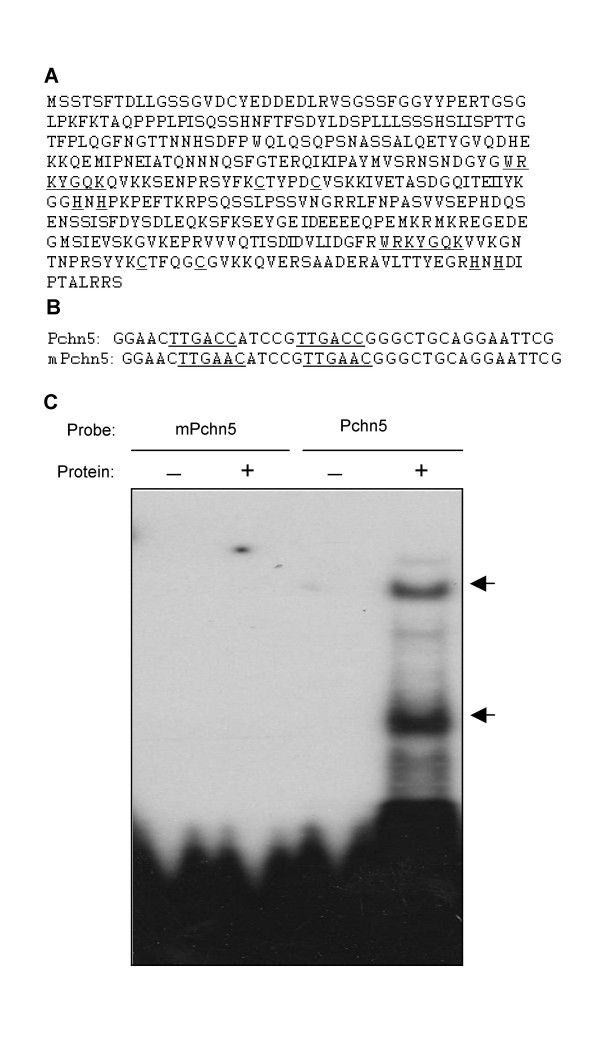
Sequence and DNA-binding Activity of WRKY25. A. Amino acid sequence of WRKY25. The two WRKY motifs are indicated with the highly conserved WRKYGQK sequence and the residues forming the C_2_H_2 _zinc fingers underlined. B. Oligonucleotides used in the electrophoretic mobility shifting assay (EMSA). The Pchn5 probe contains two direct W-box repeats, while in the mPchn5 probe, the TTGACC sequences are mutated to TTGAAC. The wild-type and mutated W-box sequences are underlined. C. EMSA to test binding of recombinant WRKY25 to the W box motif in the Pchn5 probe. Binding reactions containing WRKY25 and Pchn5 produced two major DNA/protein complexes, which are indicated by arrows. Change of the TTGACC to TTGAAC in the mPchn5 probe abolished WRKY25 binding. No retarded bands were detected in the absence of the recombinant WRKY25 protein.

*Arabidopsis WRKY25 *(At2g30250) encodes a protein of 394 amino acids with a molecular weight of 44.134 kD and an isolelectric point of 6.43 (Figure 1A). Based on the presence of two WRKY domains, WRKY25 is classified as a group I WRKY protein. The N-terminus and the region between the WRKY domains are rich in serine and/or threonine residues (Figure 1A). Thus, WRKY25 may be regulated, at least in part, via protein phosphorylation by a protein kinase(s) such as MPK4 [31].

WRKY transcription factors are thought to function by binding their cognate TTGACC/T W-box *cis*-elements in the promoter regions of target genes and activating or repressing their expression [6]. A number of isolated WRKY proteins have been shown to bind W-box sequences [8,12,20]. To examine the DNA-binding activity of WRKY25, we expressed the gene in *E. coli*, purified the recombinant protein, and assayed its binding to an oligonucleotide that contains two direct TTGACC repeats (Pchn5; Figure 1B) using EMSA. Several WRKY25/DNA complexes with differing mobility were detected when purified recombinant WRKY25 protein was incubated with the Pchn5 probe (Figure 1C). Whether the different complexes represent probes in which one or both of the W boxes are bound by WRKY25, or whether they are caused by formation of monomeric and oligomeric WRKY25 complexes is unclear. Alternatively, some of these complexes might result from protein degradation or incompletely translation of the *WRKY25 *gene. Binding of WRKY25 was not detected with a mutant probe (mPchn5) in which both TTGACC sequences were changed to TTGAAC (Figure 1B and 1C). Thus, binding of WRKY25 to the TTGACC W-box sequence is highly specific.

**Figure 2:**
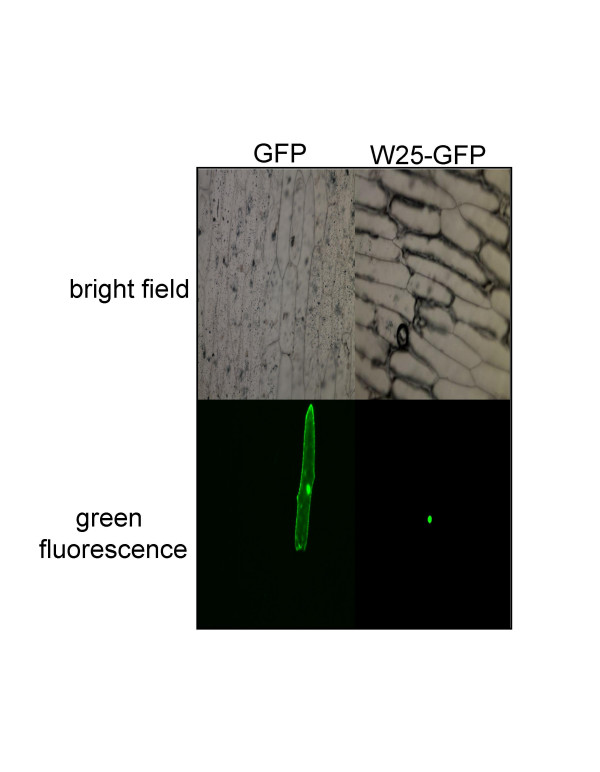
Localization of WRKY25 *in vivo*. WRKY25 was fused to GFP to yield W25-GFP; this chimeric protein was localized to the nucleus of onion epidermal cells. GFP alone was detected in both the nucleus and the cytoplasm due to its small size. Bright-field image of the onion epidermal cells are shown in the top panels.

If WRKY25 is a transcription factor, it is likely to be localized in the nucleus. The presence of putative nuclear localization signal predicted by the PSORT II program is consistent with this possibility. To determine the subcellular location of WRKY25, we constructed a GFP protein fusion of WRKY25. The fusion construct, driven by the *Cauliflower mosaic virus *(CaMV)*35S *promoter, was directly bombarded into onion (*Allium cepa*) epidermal cells. As shown in Figure 2, the transiently expressed WRKY25-GFP fusion protein was localized exclusively to the nucleus. By contrast, GFP was found in both the nucleus and cytoplasm due to its small size (Figure 2).

### Expression of *WRKY25*

**Figure 3:**
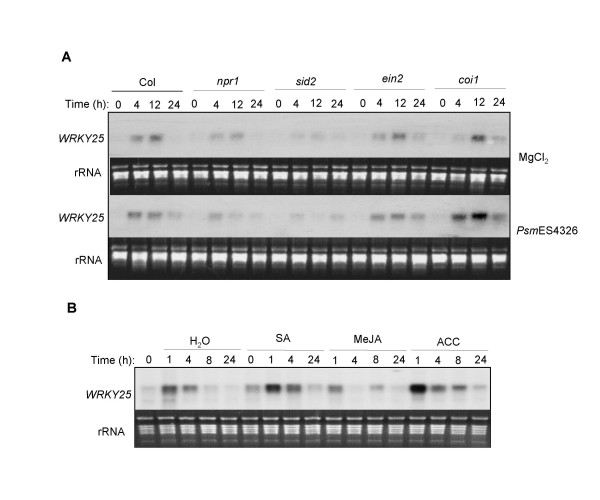
Expression of *WRKY25*. A. RNA blot analysis of *WRKY25 *expression in 5-week old wild-type or mutant *Arabidopsis*. Two fully expanded leaves were infiltrated with 10 mM MgCl_2 _(mock inoculation) or *Psm *ES4326 (OD_600 _= 0.0001 in 10 mM MgCl_2_) and harvested at the indicated times after inoculation. After separation on a gel and blotting to nylon membrane, the blot was probed with a *WRKY25-*specific DNA fragment. B. Induced WRKY25 expression in 5-week old wild-type *Arabidopsis *plants sprayed with H_2_O, 2 mM SA, 0.1 mM ACC or 0.1 mM methyl JA. Leaves were harvested at the indicated times after treatment and used for preparation of total RNA and RNA blotting. Ethidium bromide stained rRNA was used as a loading control. The experiments were repeated twice with similar results.

A possible role for WRKY25 during defense signaling was further investigated by analyzing its expression in *Arabidopsis *after inoculation with *Psm*ES4326. As shown in Figure 3A, *WRKY25 *mRNA levels increase in wild-type plants after infiltration with either the control MgCl_2 _solution (mock inoculation) or the bacterial suspension. However, *WRKY25 *expression was prolonged in pathogen-infected plants, as transcript levels remained elevated at 24 hours post infiltration (hpi), whereas they were nearly undetectable in MgCl_2_-treated plant at this time (Figure 3A).

To determine whether WRKY25 expression is influenced by the SA, ET and/or JA signaling pathways, WRKY25 expression was monitored in various signaling mutants. Induced *WRKY25 *expression was modestly reduced in the *npr1-3 *and *sid2 *mutants, which are defective in SA signaling and biosynthesis, respectively [35,36] (Figure 3A). By contrast, no significant difference was observed in *WRKY25 *expression between the wild-type plants and the ET-insensitive *ein2 *mutant plants following mock or pathogen inoculation. Analysis of the JA-insensitive *coi1 *mutant revealed a delay in *WRKY25 *expression following mock inoculation; however, it was significantly enhanced after pathogen infiltration, as compared with that observed in wild-type plants (Figure 3A). These results suggest that *WRKY25 *expression is sensitive to environmental cues and it appears to be positively regulated by the SA signaling pathway but negatively regulated by the JA pathway.

We also analyzed *WRK25 *induction in wild-type plants sprayed with water, SA, 1-aminocyclopropane-1-carboxylic acid (ACC, the immediate precursor of ET) or methyl JA. *WRKY25 *expression was rapidly induced in water-treated plants (Figure 3B), underscoring that the gene is very responsive to environmental stimuli. Plants sprayed with SA or ACC accumulated greater levels of WRKY25 transcripts than water-sprayed plants, whereas JA-treated plants accumulated less (Figure 3B). Thus, both SA and ET regulate *WRKY25 *expression in a positive manner, whereas JA has a negative effect on *WRKY25 *expression.

### Disrupting or altering *WRKY25 *expression affects disease resistance and symptom severity

**Figure 4:**
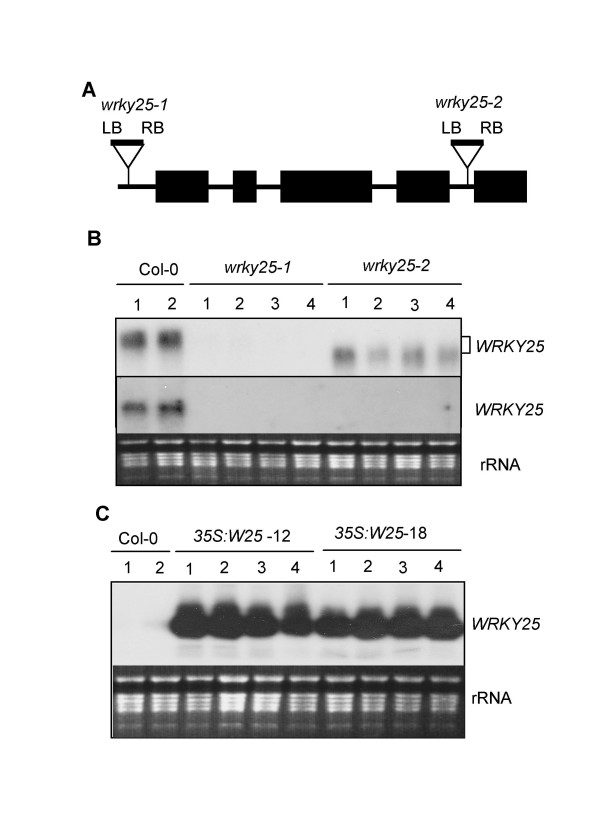
Characterization of *wrky25 *T-DNA insertion mutants and transgenic overexpressing plants. A. Diagram of *WRKY25 *gene and its T-DNA insertion mutants. B. RNA blot analysis of *wrky25 *mutant lines. Four hours after inoculation with *Psm*ES4326 (OD_600 _= 0.0001), the inoculated leaves from two wild-type plants (Col-0) or four *wrky25-1 *and *wrky25-2 *mutant plants were harvested and total RNA was isolated. After separation and blotting to a nylon membrane, the blot was probed with a full-length *WRKY25 *cDNA fragment (upper panel) or a WRKY25 DNA fragment corresponding to the region downstream of the T-DNA insertion in the *wrky25-2 *mutant (lower panel). Ethidium bromide stained rRNA was used as a loading control. C. RNA blot analysis of *WRKY25 *expression in transgenic plants constitutively overexpressing *WRKY25*. RNA samples were prepared from leaves of two wild-type plants (Col-0) or four plants from each tansgenic *35S:WRKY25 *line and probed with a *WRKY25*-specific DNA probe. Lines 12 and 18 expressed elevated levels of *WRKY25 *and contain a single T-DNA insertion based on the ratio of antibiotic-resistant progeny. F3 homozygous progeny plants of the two lines were used for further analyses.

To analyze the role of WRKY25 in disease resistance, we identified two T-DNA insertion mutants for *WRKY25*. *wrky25-1 *(Salk_136966) contains a T-DNA insertion in the promoter region while *wrky25-2 *(Sail 754_A03) contains a T-DNA insertion in the last intron of the *WRKY25 *gene (Figure 4A). Homozygous mutant plants were identified by PCR with *WRKY25*-specific primers. We then compared the wild-type and *wrky25 *mutants for Induced accumulation of *WRKY25 *transcripts. Since MgCl_2 _treatment and pathogen infection had almost the same potency in inducing *WRKY25 *expression (Figure 3), we used only pathogen infection in these experiments. Northern analysis using a full-length *WRKY25 *cDNA clone as the probe detected *WRKY25 *transcripts in wild-type plants but not in *wrky25-1 *plants after pathogen infection. By contrast, a *WRKY25 *transcript of reduced size was detected in pathogen-infected *wrky25-2 *plants (Figure 4B, upper panel). This transcript was not detected when the same blot was probed with a DNA fragment corresponding to the region downstream of the T-DNA insertion site in *wrky25-2 *(Figure 4B, lower panel). Thus, the T-DNA insertion in the *wrky25-2 *mutant results in generation of a truncated *WRKY25 *transcript that is predicted to generate a truncated WRKY25 protein lacking the C-terminal WRKY domain, which is important for DNA binding [37].

**Figure 5:**
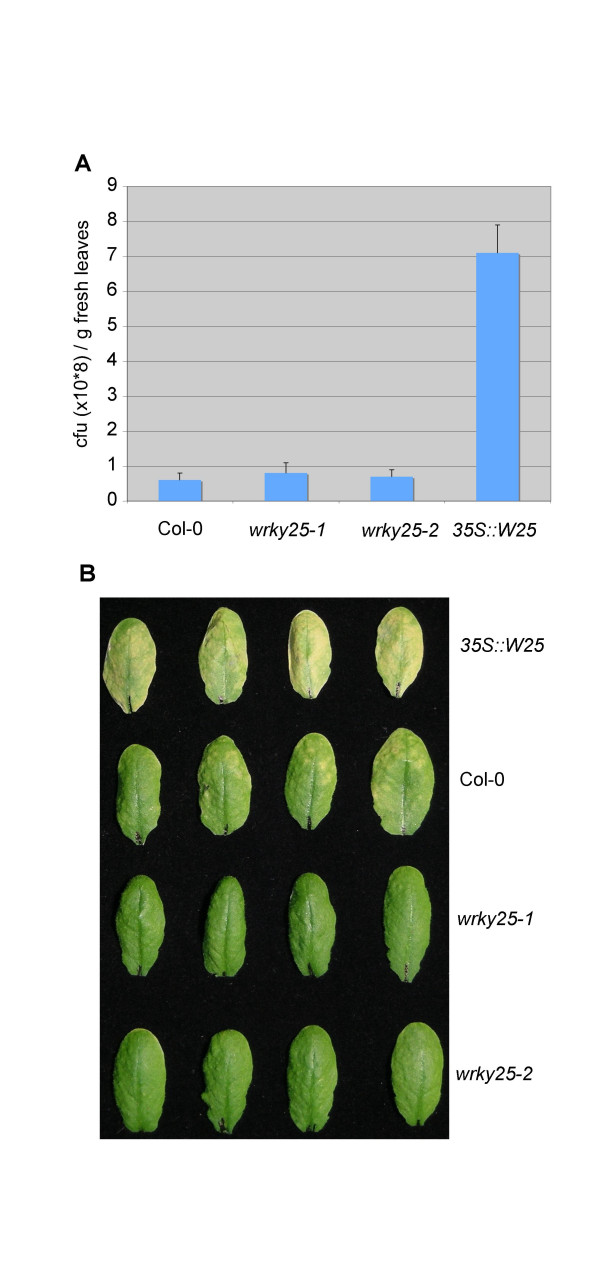
Responses of the *wrky25 *mutant and transgenic *35S:WRKY25 *plants to *P. syringae*. A. Bacterial titer in wild type (Col-0), *wrky25 *insertion mutant and F3 progeny of transgenic *35S:WRKY25 *plants (line 12) at 3 days post inoculation (dpi) with *Psm*ES4326 (OD_600 _= 0.0001). The means and standard errors were calculated from six replicates. Analysis of F3 progeny from *35S:WRKY25 *line 18 yielded similar results to those observed for *35S:WRKY25 *line 12. B. Disease symptom development in wild type (Col-0), *wrky25 *mutants and transgenic *35S:WRKY25 *plants inoculated with *Psm*ES4326 (OD_600 _= 0.0001). Pictures of representative inoculated leaves were taken at 4 dpi. These experiments were repeated two additional times with similar results.

Analysis of both *wrky25 *mutants revealed no difference in growth or morphology from that of wild-type plants; flowering also occurred at the normal time. Following inoculation with *Psm*ES4326, the mutant lines supported similar levels of bacterial growth as wild-type plants (Figure 5A). However, the inoculated leaves of *wrky25-1 *and *wrky25-2 *plants consistently displayed less disease symptom than wild-type plants (Figure 5B).

To examine the effect of *WRKY25 *overexpression, we generated plants containing a full-length *WRKY25 *cDNA driven by the *CaMV 35S *promoter (*35S::W25*). Northern blotting identified several transgenic plants that contained elevated levels of *WRKY25 *transcript constitutively (Figure 4C). Two transgenic lines (#12 and #18 in Figure 4C) that constitutively expressed *WRKY25 *at elevated levels and contain a single T-DNA locus in their genomes, based on the ratio of antibiotic resistance phenotypes, were chosen for further study.

Analysis of T_3 _homozygous plants from both lines revealed no difference in growth or development from that of wild-type plants, although their leaf color appear to be slightly paler. Following inoculation with *Psm*ES4326, the transgenic 35S:W25 overexpression lines displayed substantially greater bacterial growth (~12 fold) than wild-type plants (Figure 5A). The inoculated leaves of *WRKY25*-overexpressing plants also developed more severe disease symptoms than those of wild-type plants after infection (Figure 5B).

### *PR1 *gene expression and SA accumulation

**Figure 6:**
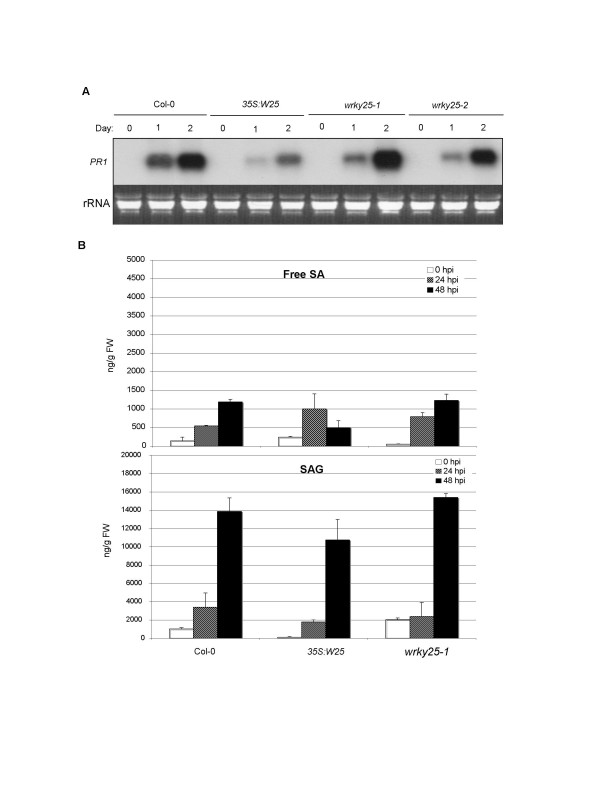
Pathogen-induced *PR1 *expression and SA accumulation. A. RNA blot analysis of PR1 expression in wild type (Col-0), F3 progeny of transgenic *35S:WRKY25 *plants (line 12) and *wrky25 *mutants following inoculation with *Psm*ES4326 (OD_600 _= 0.0001). Total RNA was isolated from inoculated leaves harvested at indicated times after inoculation and probed with a *PR1 *probe. Ethidium bromide-stained rRNA was used as a loading control. The experiment was repeated two additional times with similar results. B. Determination of free and SA glucoside (SAG) levels in wild type (Col-0), transgenic *35S:WRKY25 *plants (line 12) and the *wrky25 *mutants after inoculation with *Psm*ES4326 (OD_600 _= 0.0001). Inoculated leaves were harvested at indicated times for SA and SA glucoside (SAG) determination. The means and standard errors were calculated from 2–3 replicate samples. FW, fresh weight.

To study the molecular basis for the altered responses to *Psm*ES4326 infection, *PR1 *gene expression was monitored. Consistent with the enhanced susceptibility phenotype, *WRKY25 *overexpressing lines contained substantially lower levels of *PR1 *transcripts than wild-type plants (Figure 6A). In contrast, *PR1 *transcript levels in the *wrky25 *mutants were comparable to those in wild-type plants (Figure 6A).

To determine whether altered *PR1 *induction in the *WRKY25*-overexpressing plants correlated with reduced SA accumulation, the levels of both free SA and SA-glucoside conjugates (SAG) were monitored. Both wild-type plants and the T-DNA insertion mutants displayed similar levels of free SA and SAG following *Psm*ES4326 infection (Figure 6B). Free SA levels in *WRKY25*-overexpressing plants were comparable to those in wild-type plants at 0 and 24 hpi; however, the level of free SA at 48 hpi was somewhat lower than in wild-type plants. SAG levels in uninoculated *WRKY25*-overexpressing plants also were ~10-fold lower than those in wild-type plants, although they rose to nearly wild-type levels after infection (Figure 6B).

## Discussion and Conclusion

To determine whether WRKY25 is a transcription factor that regulates disease resistance, we analyzed its role in *Arabidopsis *responses to the bacterial pathogen *P. syringae*. Consistent with this putative function, WRKY25 is a nuclear-localized, DNA-binding protein that specifically recognizes the TTGAC W-box sequences (Figures 1 and 2). Analysis of WRKY25 expression revealed that it is highly responsive to a variety of biotic and abiotic stress conditions (Figure 3). In wild-type plants, pathogen infection induced prolonged *WRKY25 *expression, whereas induction by mock inoculation was more transient. SA treatment also induced *WRKY25 *expression over that observed in water-treated plants, whereas it was reduced in JA-treated plants. Further arguing that the SA and JA signaling pathways play positively and negatively roles in regulating *WRKY25 *induction, respectively, was the demonstration that pathogen-induced *WRKY25 *expression was reduced in *npr1-3 *and *sid2 *mutants and enhanced in the *coi1 *mutant. Although JA frequently works with ET to signal defense responses, the role of ET in *WRKY25 *expression is unclear. ACC treatment induced *WRKY25 *expression, but pathogen-induced expression of this gene was unaffected in the *ein2 *mutant. Taken together, these results indicate that multiple signaling pathways associated with stress responses influence *WRKY25 *induction.

Strong evidence that this stress-induced *WRKY *gene functions as a negative regulator of defense against *P. syringae *comes from analysis of both transgenic overexpression lines and two T-DNA insertion mutants. Constitutive overexpression of *WRKY25 *suppressed pathogen-induced *PR1 *expression and enhanced both bacterial growth and symptom development as compared with wild-type plants, whereas the *wrky25 *mutants displayed reduced disease symptoms. Since the *wrky25-1 *and *wrky25-2 *mutants supported wild-type levels of bacterial growth, *PR1 *expression and SA accumulation, it is possible that WRKY25 exerts its negative effect(s) primarily by promoting disease symptom development. Consistent with this possibility, several studies have suggested that bacterial growth and symptom development are not necessarily linked and can be differentially affected by host genes. For example, the ET-insensitive *ein2 *mutant supports normal bacterial growth but develops reduced chlorosis after infection by *P. syringae *as compared to wild-type plants [38]. In addition, disruption of the *Arabidopsis BOTRYTIS-SUSCEPTIBLE 1 *(*BOS1*) gene, which encodes a R2R3 MYB transcription factor, causes enhanced disease symptoms after infection of *P. syringae *without affecting bacterial growth [39].

Alternatively, the observation that *WRKY25*-overexpressing plants displayed increased bacterial growth and enhanced disease symptoms suggests that this protein promotes both processes. If so, one possible explanation for why bacterial growth was unaffected in the *wrky25 *mutants is that WRKY25 function might vary depending on its expression level and/or biological context. Thus, relatively low-level and possibly tissue-specific expression of *WRKY25*, as in the wild-type plants, might promote symptom development, whereas the high-level, non-specific expression, as in the overexpression lines, might promote both disease symptoms and bacterial growth. Alternatively, functional redundancy between various members of the large WRKY gene family might mask the effect of a mutation in a single WRKY gene. WRKY25 shares very similar protein structure with WRKY33 and they also display similar pathogen-induced expression patterns [13,40]. To test possible functional redundancy between the two WRKY proteins, we have generated double knockout mutants for the two genes but observed no further enhancement in the phenotypes of disease resistance to either necrotrophic fungal pathogens or *P. syringae *(Zheng and Chen, unpublished results).

A growing body of evidence indicates that pathogen infection can activate multiple signaling pathways in the regulation of diverse defense mechanisms [4,41]. SA-mediated signaling pathway(s), for example, play important roles in defense against biotrophic pathogens and in activation of systemic acquired resistance against a diverse range of microbial pathogens [41]. Defense pathways mediated by JA and/or ET, on the other hand, are more effective against herbivores and necrotrophic pathogens [41]. While these distinct defense pathways can sometimes function cooperatively, they often are antagonistic. SA has been shown to play an important role in signaling resistance to *P. syringae *[34,35,42]. Thus, one possible mechanism through which WRKY25 negatively regulates defense responses after *P. syringae *infection is by repressing the SA signaling pathway. Supporting this possibility, plants overexpressing *WRKY25 *displayed reduced pathogen-induced expression of *PR1*, a molecular marker for SA-mediated defense pathways. Since these overexpressing plants accumulated nearly wild-type levels of free SA, WRKY25 may work at a point downstream of SA to repress defense responses. Previous studies have revealed that suppression of the SA signaling pathway enhances the activation of JA-induced responses. Thus, we tested whether *WRKY25 *overexpressing lines and/or *wrky25 *mutants displayed altered responses to two necrotrophic fungal pathogens, *Botrytis cinerea *and *Alternaria brassicicola*. Neither the *WRKY25 *overexpression lines nor the mutants lines displayed altered fungal growth or symptom development (unpublished results).

It was recently demonstrated that WRKY25 is phosphorylated by MPK4 *in vitro*; WRKY25 also interacts with MKS1, a substrate of MPK4 [31]. These results, together with our analysis, suggest that WRKY25 functions in the MPK4 signaling pathway that represses SA-mediated defense responses. WRKY33 appears to function largely as an activator of JA/ET-mediated signaling [32]. These studies support that these two WRKY transcription factors function in the MPK4-mediated SA-repressing and JA/ET-activating signaling pathways. In addition, our analyses revealed that *WRKY25 *expression is induced by abiotic stress, such as infiltration with MgCl_2 _or spraying with water. Previous studies have demonstrated that *WRKY25 *also is induced by oxidative stress, heat shock and wounding [43]. Thus, this transcription factor may play a role in responses to abiotic as well as biotic stresses. However, transgenic plants constitutively expressing *WRKY25 *were not more tolerant to oxidative stress, as measured by root growth on agar plates in the presence or absence of the superoxide-generating agent paraquat [43]. Whether our *WRKY25 *overexpression plants and/or the loss-of-function mutants exhibit altered responses to other abiotic stress conditions has not been determined. Future studies in this direction may reveal not only additional roles for WRKY25 in plant defense and stress responses, but also possible interactions between their respective signaling pathways.

## Methods

### Materials

[α-^32^P]dATP (>3,000 Ci/mmol) was obtained from New England Nuclear; other common chemicals were purchased from Sigma. *Arabidopsis *plants were grown in growth rooms at 22°C and 120 μEm^-2^s^-1 ^light on a 12 hour light and 12 hour dark photoperiod. SA was dissolved in water as a 100 mM stock solution and adjusted to pH 6.5 with KOH. SA was applied by spraying the plants with a 2 mM solution. MeJA was dissolved in 50% ethanol as a 10 mM stock solution and diluted to 100 μM with water before spraying onto plants. ACC was dissolved in water as a 10 mM stock solution and was diluted to 100 μM before spraying the plants.

### Northern blotting

Total RNA was isolated from leaves using the TRIZOL reagent (BRL Life Technologies, Rockville, MD). For RNA gel blot analysis, total RNA (4 μg) was separated on 1.2% agarose-formaldehyde gels and blotted to nylon membranes. Blots were hybridized with [α-^32^P]dATP labeled gene-specific probes. Hybridization was performed in PerfectHyb plus hybridization buffer (Sigma) overnight at 68°C. The membrane was then washed for 10 minutes twice with 2× SSC (1× SSC is 0.15 M NaCl and 0.015 M sodium citrate) and 1% SDS and for 10 minutes with 0.1× SSC and 1% SDS at 68°C. Transcripts for *WRKY25 *were detected with a full-length *WRKY25 *cDNA as probe unless otherwise indicated. Transcripts for *PR1 *were detected using a *PR1 *fragment generated from PCR amplification using two *PR1*-specific oligonucleotides as primers (5'-TTCTTCCCTCGAAAGCTCAA-3' and 5'-CGTTCACATAATTCCCACGA-3').

### Production of recombinant WRKY25 protein and electrophoresis mobility shift assay (EMSA)

To generate the WRKY25 recombinant protein, its full-length cDNA was amplified using two gene-specific primers (5'-ATCGAATTCATGGACAATAGCAGAAC-3' and 5'-ATCCTCGAGTGAGGGCATAAACGAAT-3'). The amplified DNA fragment was digested with EcoR1 and Xho1, cloned into the same sites of pET32a (Novagen) and transformed into *Escherichia coli *strain BL21(DE3). Induction of expression and purification of recombinant His-tagged WRKY25 protein were performed according to the protocol provided by Novagen. The purified proteins were dialyzed overnight against a nuclear extract buffer (25 mM HEPES/KOH at pH 7.5, 40 mM KCl, 0.1 mM EDTA, 10% glycerol, 1 mM DTT and 30 mg ml/l PMSF) at 4°C. The double-stranded synthetic oligonucleotide Pchn5 was designed based on the sequence from the promoter of the tobacco basic chitinase gene [18] with two W boxes separated by 5 nucleotides. Pchn5 and the mutant mPchn5 probes were labeled to specific activities of approximately 10^5 ^cpm/ng using the Klenow fragment of DNA polymerase I. Sequence-specific DNA binding was assayed with EMSA essentially as described previously [12]. Binding reactions contained 12 μl nuclear extraction buffer, 5 μg poly(dIdC), 1 μg proteins and 2 ng of labeled double-stranded oligo DNA. DNA and protein complexes were allowed to form at room temperature for 30 min and resolved on a 10% polyacrylamide gel in 0.5× TBE at 4°C.

### Subcellular localization

Full length *WRKY25 *coding sequence was amplified with two gene-specific primers (5'-ATCGAATTCATGGACAATAGCAGAAC-3' and 5'-ATCCCATGGTGGGCATAAACGAATCG-3'). The amplified fragment was digested with EcoR1 and Nco1 and cloned into a GFP vector behind the *CaMV 35S *promoter. The empty GFP plasmid was used as a control. Transient expression of the GFP fusion proteins in onion epidermal cells through particle bombardment of the GFP construct plasmid DNA was performed essentially as described [44].

### Construction of transgenic lines

To generate the *35S:WRKY25 *construct, the cDNA fragment that contained the full coding sequence and 3' untranslated region of *WRKY25 *was excised from a cloning plasmid and subcloned into the same restriction sites of the *Agrobacterium *transformation vector pOCA30 [27] in the sense orientation behind the *CaMV 35S *promoter. *Arabidopsis *transformation was performed by the floral dip procedure [45]. The seeds were collected from the infiltrated plants and selected in MS medium containing 50 μg/ml kanamycin. Kanamycin-resistant plants were transferred to soil nine days later and grown in a growth chamber.

### Identification of the *wrky25 *T-DNA insertion mutants

The T-DNA insertion flanking sequences of the Syngenta *Arabidopsis *insertion library and Salk T-DNA insertion population [46] were searched with the genomic sequence of *WRKY25*. Confirmation of the T-DNA insertions was done by PCR analysis using a combination of a gene specific primer (5'-CGGTTTCACACTTGACGATTT-3' for *wrky25-1 *and 5'-GCAAAAGGTTTCTTCTTGGGT-3' for *wrky25-2*) and a T-DNA border primer (5'-GCTTGCTGCAACTCTCTCAG-3' for *wrky25-1 *and 5'-TAGCATCTGAATTTCATAACCAATCTCGATACAC-3' for *wrky25-2*). The nature and locations of the T-DNA insertions were confirmed by sequencing the PCR products. Another PCR was performed to identify plants homozygous for the T-DNA insertions using the above gene-specific primers and respective reverse primers (5'-AGACCCGGTTCTCTCTGGAT-3' for *wrky25-1 *and 5'-TCACGAGCGACGTAGCGCGGT-3' for *wrky25-2*).

### Pathogen infection

Pathogen inoculation was performed by infiltrating the leaves of at least six plants for each treatment with either 10 mM MgCl_2 _(mock inoculation) or a suspension of the bacterial pathogen *P. syringae. Psm*ES4326 was cultured until OD_600 _= 0.6–1 in liquid King's medium B with an appropriate antibiotic at 28°C. After collecting the cells by centrifugation, the cells were re-suspended in 10 mM MgCl_2 _and adjusted to appropriate concentrations for plant infiltration. To assess bacterial growth, leaves were weighed, homogenized in 10 mM MgCl_2 _and appropriate dilutions were plated onto King's medium B agar supplemented with appropriate antibiotics. After incubation for 48 hours at 28°C, the number of colony-forming units (cfu) per gram of infected leaf tissue was determined.

### Determination of SA

Free SA and SAG were extracted and quantified as described previously [47].

## Authors' contributions

ZZ carried out the molecular characterization of the WRKY25 protein and functional analysis of the mutants and drafted the manuscript. SLM carried out quantification of SA. BF carried out isolation of the mutants and participated in their characterization. DFK participated in the design of the study and edited the manuscript. ZC conceived of the study, participated in the design and helped to draft and edit the manuscript. All authors read and approved the final manuscript.
